# Association Between Serum Folate Levels and Caffeinated Beverage Consumption in Pregnant Women in Chiba: The Japan Environment and Children’s Study

**DOI:** 10.2188/jea.JE20170019

**Published:** 2018-10-05

**Authors:** Masae Otake, Kenichi Sakurai, Masahiro Watanabe, Chisato Mori

**Affiliations:** 1 Center for Preventive Medical Sciences, Chiba University, Chiba, Japan; 2 Department of Environmental Medicine, Graduate School of Medicine, Chiba University, Chiba, Japan

**Keywords:** pregnancy, folate, caffeine, consumption, tannin (catechin)

## Abstract

**Background:**

Several studies have reported the adverse effects of caffeine intake during pregnancy on fetal health. However, the effects of caffeine intake from green and oolong teas has not been investigated, despite the considerable consumption of these teas in Japan and the potential inhibitory effects of catechins—chemicals present at relatively high levels in green and oolong teas—on folic acid absorption. The potential associations of serum folate levels with caffeinated beverage consumption and catechin levels remain largely unstudied. The present study aimed to determine these associations in pregnant Japanese women.

**Methods:**

Pregnant women (*n* = 2,701) not receiving folate supplementation were enrolled at the Chiba Unit Center, a regional site of the Japan Environment and Children’s Study (JECS). Serum folate levels were measured using an Access folate assay kit, and nutrient and caffeine intakes were assessed using a self-administered food frequency questionnaire that was previously evaluated in Japanese populations.

**Results:**

The low and normal serum folate groups reported caffeine intakes of 42.3 mg/1,000 kcal and 34.4 mg/1,000 kcal, respectively, and tannin intakes of 40.8 mg/1,000 kcal and 36.3 mg/1,000 kcal, respectively. Multiple regression analyses revealed negative associations of serum folate levels with caffeine and tannin intakes and a positive association between serum folate levels and dietary folate intake.

**Conclusions:**

Considering the negative associations of caffeine and tannin levels with serum folate levels, pregnant women should consume caffeinated beverages, such as coffee and green/oolong teas, with caution.

## INTRODUCTION

Humans cannot synthesize folate, an essential water-soluble vitamin involved in DNA synthesis and cell differentiation. Prenatal folate deficiency, particularly during early pregnancy, increases the risk of neural tube defects (NTDs), such as spina bifida and anencephaly.^1^ In addition, a low maternal serum folate level increases the infant’s risk of small-for-gestational-age (SGA) and low-birth-weight (LBW) status.^2^^–^^4^ In Japan, the incidence of NTDs has not changed since 2003 and remains higher than that in other developed countries.^5^ Therefore, the Japanese Dietary Reference Intakes (2010) recommends a daily dietary folate intake of 400 µg for women who plan to or may become pregnant.^6^

Low serum folate levels may be attributed to several causes. First, the insufficient consumption of foods containing B vitamins, such as folate, can make it difficult to maintain adequate levels. Although dietary intake is the primary source of folate, the traditional Japanese dietary pattern has become Westernized in recent years, leading to annual decreases in vegetable consumption.^7^ Second, folate is easily excreted from the body, so folate levels are not maintained in vivo over long periods. Additionally, folate is unstable and has a low bioavailability. Because folate present in vegetables and fruits is easily destroyed by cooking or preservation techniques, approximately only 50% is utilized by the body.^8^

Several studies have suggested that maternal caffeine consumption via coffee, black tea, or soft drinks during pregnancy has several potential negative fetal effects, including NTDs, LBW, and SGA.^9^^,^^10^ However, other studies have not reported an increased risk of these fetal effects following maternal caffeine consumption.^11^^,^^12^ Studies that have examined serum homocysteine levels in association with caffeine consumption have indirectly suggested an association between caffeine intake and serum folate levels.^13^^,^^14^ For example, a recent study reported a positive association between the consumption of coffee, which contains high levels of caffeine, and serum concentrations of homocysteine,^15^ a potential indicator of a reduced folate level. Therefore, the guidelines established by the World Health Organization (WHO) and international food standard and food safety agencies indicate that pregnant women should limit the intake of caffeinated beverages to 3–4 cups a day.^16^ In 2011, the Food Safety Commission of Japan announced their intent to gather information and determine provisions regarding caffeine intake via coffee.^17^ In 2014, the Guidelines for Obstetrical Practice in Japan^18^ noted the potential risk of fetal growth restriction from caffeine abuse and suggested that pregnant Japanese women should limit their daily caffeine intake to 300 mg (eg, 3 cups of coffee).

In the United States and Europe, coffee and black tea are the main beverage sources of caffeine; as a result, estimations of the effects of caffeine intake on folate status have relied only on these sources.^19^^–^^21^ Given the high rate of green and oolong tea consumption in Japan,^22^ however, the effects of caffeine intake from green and oolong teas on folate status should be investigated. In addition to caffeine, green and oolong teas contain relatively high amounts of catechins, which can inhibit folic acid absorption.^23^ Notably, studies of the association between maternal tea intake during pregnancy and serum folate levels have been conducted in Japan (*n* = 254)^24^ and China (*n* = 1,724).^25^ In the former study, serum folate levels were lower among subjects who consumed large amounts of green and oolong teas, and the authors suggested that catechins might affect folate absorption. The latter study, however, failed to find an association between tea (green, oolong, black, and jasmine) intake and folate levels.^25^ Despite the existence of these few studies, research concerning the associations of serum folate with caffeinated beverage intake (including green and oolong teas) and catechin levels remains limited. Therefore, the present study aimed to determine these associations in a Japanese population of pregnant women.

## MATERIALS AND METHODS

### Subjects and definitions

This research used the first-year fixed dataset of the Japan Environment and Children’s Study (JECS), which was initiated by the Ministry of the Environment in Japan as a nationwide birth cohort to investigate the associations of environmental factors with aspects of children’s health and development.^26^^,^^27^ The Chiba Unit Center, a regional JECS site, recruited expecting women for 3 years (2011–2014) and enrolled those who agreed to participate before delivery (*n* = 4,207). Subjects for whom serum folate or food frequency questionnaire (FFQ) data during the third trimester were unavailable, and those receiving folic acid supplementation, were excluded from this analysis. We also excluded subjects with significantly under- (intake ≤600 kcal or ≤0.5 times Group I, low physical activity level) or over-reported FFQ energy intakes (≥4,000 kcal or ≥1.5 times Group III, high physical activity level), based on the “Overview of Dietary Intakes for Japanese”^6^^,^^28^ (*n* = 2,701). The third trimester was defined as gestational week 22 and beyond.

### Serum folate analysis and food frequency questionnaire

Blood samples were collected during the third trimester. Serum folate levels were measured using an Access folate assay kit (FOLW; SRL Co., Ltd., Tokyo Japan).^29^^,^^30^ The WHO Technical Consultation suggests that folate deficiency be defined as a serum folate <10 nmol/L (<4 ng/mL), based on the United States National Health and Nutrition Examination Survey III.

The FFQ was completed during the third trimester. This tool, which was used in the JECS, is a validated, self-administered diet questionnaire that was previously evaluated in Japanese studies concerning nutritional factors.^31^^–^^35^ The subjects reported their dietary intakes and meal frequencies, beginning with their first indication of pregnancy. The nutrient and food intake amounts were energy-adjusted using the energy density method: the percentages of total energy intake (%E) were calculated for protein, fat, and carbohydrates; other items were reported using the intakes per 1,000 kcal total energy. Caffeine in beverages (coffee and black, green, and oolong teas) was measured using the total daily amount (per 1,000 kcal). The total amounts of caffeine and tannins in coffee and black, green, and oolong teas were calculated based on the 2014 Standard Tables of Food Composition in Japan.^6^

Green and oolong teas contain (−)-epigallocatechin gallate, which interferes with folate metabolism.^36^^,^^37^ Therefore, we considered both beverages to contain catechins and categorized them in a single group (green/oolong tea) for this analysis. Because we could not directly estimate catechin amounts using the 2014 Standard Tables of Food Composition in Japan,^6^ we substituted tannin intake for the estimated catechin intake, as tannins comprise 80–85% catechins.

### Analysis

The statistical analysis was conducted using SPSS version 23 (IBM Corp., Armonk, NY, USA), R (ver. 3.1.10; R Project for Statistical Computing, Vienna, Austria), and Excel Statistics (Microsoft Co., Ltd., Tokyo, Japan). Statistical significance was set at 5%.

To investigate the associations of serum folate levels with the dietary intake of each food or nutrient, subjects were divided into two groups based on folate levels: normal folate, defined as ≥4.0 ng/mL (*n* = 1,980), and low folate, defined as <4.0 ng/mL (*n* = 721). The chi-square and Mann–Whitney U tests were used for inter-group comparisons.

Two different multiple regression analyses models were used to investigate the effects of caffeine and tannin intakes separately on maternal serum folate levels. One model used serum folate as a dependent variable and age, dietary folate, and total caffeine as independent variables. The other used serum folate as a dependent variable and age, dietary folate, and tannin as independent variables.

## RESULTS

The baseline characteristics of pregnant subjects in this Japanese study population (*n* = 2,701) were as follows: subjects had a median age of 30 (interquartile range [IQR], 27–34) years, median height of 158.0 (IQR, 154.7–162.0) cm, and median pre-pregnancy weight of 52.0 (IQR, 48.0–58.0) kg. The median serum folate level was 5.3 (IQR, 3.8–7.8) ng/mL, the median daily energy intake was 1633 (IQR, 1,332–2,047) kcal, and the median daily dietary folate intake was 235 (IQR, 174–322) µg/1,000 kcal. The median caffeine intakes from coffee, black tea, and green/oolong tea were 9.1 (IQR, 0–34.7) mg/1,000 kcal, 3.8 (IQR, 0–10.7) mg/1,000 kcal, and 9.2 (IQR, 0–22.3) mg/1,000 kcal, respectively; corresponding tannin intakes were 3.8 (IQR, 0–14.5) mg/1,000 kcal, 1.3 (IQR, 0–3.6) mg/1,000 kcal, and 22.9 (IQR, 0–55.9) mg/1,000 kcal, respectively.

In this cohort, 73.3% (*n* = 1,980) of the subjects had serum folate levels ≥4.0 ng/mL (normal-folate group), and 26.7% (*n* = 721) had levels <4.0 ng/mL (low-folate group). Compared with those with normal folate, subjects with low folate had a lower mean age and higher numbers of births and previous gravidity (Table 1). Subjects in the normal-folate group also had a lower pre-pregnancy body mass index. The groups did not differ significantly regarding educational background or annual income.

**Table 1.** Comparison of the characteristics, food intakes, serum folate levels, and caffeine or tannin intakes between pregnant Japanese women in the low-folate and normal-folate groups (*n* = 2,701)

		Serum folate <4.0 ng/mL		Serum folate ≥4.0 ng/mL		*P*-value
		*n* = 721		*n* = 1,980		
		Median (1st–3rd)		Median (1st–3rd)		
Age, years		30 (27–34)		31 (27–34)		0.016^a^
Pre-pregnancy BMI, kg/m^2^		21.0 (19.5–23.2)		20.7 (19.2–22.8)		0.021^a^

Previous gravidity^c^	0	141	19.6%	573	28.9%	<0.001^b^
	1	243	33.7%	698	35.3%	
	2	182	25.2%	402	20.3%	
	3	88	12.2%	158	8.0%	
	4	27	3.7%	65	3.3%	
	≥5	25	3.5%	42	2.1%	
	Missing data	15	2.1%	42	2.1%	

Number of births^c^	0	184	25.5%	738	37.3%	<0.001^b^
	1	323	44.8%	787	39.7%	
	2	165	22.9%	329	16.6%	
	≥3	32	4.4%	72	3.6%	
	Missing data	17	2.4%	54	2.7%	

Education level^c^	High school and below	334	46.3%	767	38.7%	0.002^b^
	Junior/technical college	273	37.9%	819	41.4%	
	College/university	113	15.7%	383	19.3%	
	Missing data	1	0.1%	11	0.6%	

Income, yen/year^c^	<2,000,000	28	3.9%	75	3.8%	ns^b^
	2,000,000 to <8,000,000	600	83.2%	1,606	81.1%	
	≥8,000,000	60	8.3%	219	11.1%	
	Missing data	33	4.6%	80	4.0%	

Number of people in the household	0	2	0.1%	5	0.1%	ns^b^
	1	323	44.8%	898	45.4%	
	2	312	43.3%	840	42.4%	
	3	41	5.7%	98	4.9%	
	≥4	16	2.2%	69	3.5%	
	Missing data	27	3.7%	70	0.4%	

Serum folate, ng/mL		3.1 (2.7–3.5)		6.4 (5.0–9.0)		<0.001^a^

Energy, kcal/day		1,622 (1,306–2,033)		1,676 (1,351–2,055)		0.092^a^
Dietary folate, µg/1,000 kcal		214 (157–304)		241 (181–328)		<0.001^a^

Total caffeine intake, mg/1,000 kcal		42.3 (19.5–75.7)		34.4 (13.9–66.6)		<0.001^a^
Coffee caffeine		11.2 (0.0–39.5)		8.5 (0.0–32.7)		0.012^a^
Black tea caffeine		5.2 (0.0–12.1)		3.3 (0.0–10.3)		0.002^a^
Green/oolong tea caffeine		9.2 (0.0–23.4)		9.0 (0.0–22.2)		0.289^a^
Total tannin intake, mg/1,000 kcal		40.8 (17.4–79.2)		36.3 (13.3–72.3)		<0.001^a^
Coffee tannin		4.7 (0.0–16.5)		3.5 (0.0–13.6)		0.012^a^
Black tea tannin		1.7 (0.0–4.0)		1.1 (0.0–3.4)		0.002^a^
Green/oolong tea tannin		23.1 (0.0–58.5)		22.5 (0.0–55.5)		0.289^a^

BMI, body mass index; ns, not significant.

The significance level was set at a *P* value of <0.05 and determined using ^a^the Mann–Whitney test or ^b^chi-square test.

^c^Number of participants and percentages (%) for Previous gravidity, Number of births, Education level, Income, and Number of people in the household.

Although the groups did not differ with respect to energy intake, the normal-folate group had a higher dietary folate intake, compared to the low-folate group. The groups did not differ regarding the consumption of potatoes, sugar, fish, eggs, and oil, whereas the normal-folate group consumed significantly smaller amounts of grain, fruits, and meats and significantly larger amounts of beans, nuts, vegetables, mushrooms, seaweed, and dairy products than the low-folate group. The total caffeine intake of the low-folate group was significantly higher than that of the normal-folate group (42.3 mg/1,000 kcal vs 34.4 mg/1,000 kcal, *P* < 0.001). Similarly, the low-folate group had a significantly higher total tannin intake compared to the normal-folate group (40.8 mg/1,000 kcal vs 36.3 mg/1,000 kcal, *P* < 0.001).

The multiple regression analyses identified age, dietary folate, total caffeine intake, and total tannin as significant variables (Table 2). A negative association was observed between serum folate levels and total caffeine intake, whereas a positive association was observed between serum folate levels and dietary folate intake. Total tannin intake was also identified as a negative variable.

**Table 2.** Multiple regression analysis of the effects of variables on serum folate levels in pregnant Japanese women (*n* = 2,701)

			95% CI	
Variables	Standardized beta	*P*-value	Lower bound	Upper bound
Age, years	0.074	<0.000	0.044	0.104
Dietary folate, µg/1,000 kcal	0.002	0.002	0.001	0.003
Total caffeine intake, mg/1,000 kcal	−0.007	<0.000	−0.010	−0.004

			95.0% CI	
Variables	Standardized beta	*P*-value	Lower bound	Upper bound

Age, years	0.069	<0.000	0.038	0.099
Dietary folate, µg/1,000 kcal	0.002	0.001	0.001	0.003
Total tannin intake, mg/1,000 kcal	−0.004	<0.000	−0.006	−0.002

CI, confidence interval.

Standardized beta indicates the standardized coefficient.

## DISCUSSION

In this study, we investigated the association of caffeine or tannin intake with serum folate levels during pregnancy. Briefly, subjects in the low-folate group had higher total caffeine and tannin intakes compared to those in the normal-folate group. Through multiple regression analyses, we found that serum folate level associated positively with dietary folate intake and negatively with caffeine and tannin intake. Serum folate levels decreased with increasing caffeine intake, despite concomitant increases in dietary folate intake. In this study, coffee, green/oolong tea, and black tea accounted for approximately 47%, 38%, and 15% of the total caffeine intake, respectively. By contrast, most of the total tannin intake, about 80%, was attributed to green/oolong tea, followed by coffee at 16% and black tea at 4%.

### Effects of caffeine on serum folate levels

Our data suggest that caffeine intake negatively influences serum folate levels. As mentioned above, coffee and green/oolong tea were the main sources of dietary caffeine during pregnancy. We note that a 100-g serving of green tea contains approximately one-third of the caffeine present in an equivalent serving of coffee. However, heavy green/oolong tea consumption can contribute to increased caffeine intake. Therefore, the frequent consumption of green and oolong teas in Japan necessitates the consideration of these beverages as caffeine sources.

### Possible role of caffeine metabolism in serum folate level decreases

The observed relationships among caffeine intake, serum folate levels, and dietary folate intake suggest that intake of caffeinated beverages negatively influences serum folate levels. Although the underlying biological mechanism remains uncertain, there are two possible explanations regarding the negative effects of caffeine on folate levels. First, caffeine stimulates the central nervous system and acts as a diuretic, thus increasing the rate of elimination of water-soluble B vitamins, including folate.^38^ Second, caffeine may affect the metabolism of homocysteine, an intermediate product of methionine recycling in the liver via a process that requires (and ultimately consumes) folate and the vitamins B_2_, B_6_, and B_12_.^39^ Caffeine is responsible for the homocysteine-increasing effects of coffee.^40^ These data suggest that a high caffeine intake would affect the bioavailability of folic acid and reduce serum folic acid levels.

### Possible interference of catechins

Multiple regression analyses were performed to investigate the influence of catechins on serum folate levels, using tannin intake as a surrogate variable. Our analysis suggests a negative association between serum folate levels and total tannin intake. Previous studies reported that green and oolong teas, which contain catechins, could inhibit folic acid absorption.^23^ Dietary folate is converted to dihydrofolic acid by folate reductase and to tetrahydrofolate (THF) by dihydrofolate reductase (DHFR). Upon methylation, THF converts to 5-methyl THF. In summary, dietary folate is taken up by cells as 5-methyl THF. EGCG-containing teas (eg, green and oolong teas) inhibit DHFR, resulting in decreased circulating folate levels.^8^ According to experiments conducted in rats, green tea catechin intake reduced serum 5-methyl THF concentrations. By contrast, regular green tea consumption was considered unlikely to affect the folate statuses of healthy men.^41^ Our data revealed a negative association between serum folate level and total tannin intake, results which differed from the findings of an earlier human study. This difference may be attributable to multiple factors. For example, our participants had a daily folate intake of approximately 240 µg, or less than that reported in the previous study (>300 µg/day). Additionally, our subjects were pregnant Japanese women who were recommended to intake 480 µg of dietary folate per day; accordingly, the reported intake was not sufficient for their situation. These factors might have affected the results of our study. Furthermore, differences in the gut microbiota of British and Japanese subjects^42^ might alter the effects of catechins. Further studies are needed to clarify the effects of catechins on human serum folate levels.

In Japan, the guidelines regarding caffeine consumption among pregnant women address only coffee.^17^ However, the caffeine in green tea might also be associated with the risks of preterm birth (PTB)^43^ and spina bifida.^44^ Maternal caffeine intake in Japan and China, which mainly occurs via tea, has been associated with a greater risk of PTB. Although the data did not support an overall association between tea consumption and spina bifida, the antifolate properties of tea suggest a potential interaction between tea intake and folic acid intake. Green tea and black tea extracts might influence the rate of folate absorption.^22^ Therefore, the intake of caffeinated green and oolong teas should be a matter of concern and underscores the need for conservative guidelines and awareness campaigns for pregnant women.

Several studies have reported a positive association between serum folate levels and dietary folate intake,^45^^,^^46^ and vegans were found to have higher serum folate levels when compared with non-vegetarians.^47^^–^^49^ Our results were consistent with those of previous studies and support the strong recommendation of an adequate intake of folate-containing foods by pregnant women.

This study had some limitations. First, the FFQ was self-administered, and the types of green tea were not clearly delineated. Although the FFQ has been investigated and reported to be valid, it is difficult to calculate the true intakes of foods and nutrients. Second, soft drinks were not included as caffeinated beverages in this study, although some (eg, cola) may contain caffeine. Although the FFQ included a “carbonated drinks” item, it did not specify the type of carbonated drink. Third, green teas were not specified by type (eg, jasmine or *hoji* tea). Accordingly, it was difficult to precisely estimate tannin consumption. In general, although the green tea type and steeping time will affect the tannin (catechin) concentration, the differences with respect to tea type and steeping time were thought to be small and negligible.

In conclusion, this study of the association between caffeinated beverage consumption and serum folate levels found that the folate tended to decrease with increasing intake of caffeinated beverages, despite increases in dietary folate intake. Therefore, the consumption of caffeinated beverages is negatively associated with serum folate levels during pregnancy. Pregnant women should abstain from drinking large quantities of caffeinated beverages, such as green tea, oolong tea, and coffee.
